# Osteochondrogenesis derived from synovial fibroblasts in inflammatory arthritis model

**DOI:** 10.1186/s41232-020-00115-w

**Published:** 2020-05-01

**Authors:** Yoko Miura, Satoshi Kanazawa

**Affiliations:** Department of Neurodeveopmental Disorder Genetics, Nagoya City University Graduate School of Medical Sciences, 1 Kawasumi, Mizuho-cho, Mizuho-ku, Nagoya, 467-8601 Japan

**Keywords:** Rheumatoid arthritis, Pannus, Boney ankylosis, Endochondral ossification, Hypertrophic chondrocyte

## Abstract

Rheumatoid arthritis (RA) is characterized by chronic joint inflammation, which forms pannus with bone destruction. Bony ankylosis is also observed following inflammation; however, the mechanism behind this aberrant bone formation in RA had remained unclear. Based on our recent findings obtained using a novel arthritis model called D1BC mouse, we found that synovial fibroblasts in pannus consist of at least three different populations with the osteochondrogenic lineage being predominant. We also found endochondral ossification like that in embryonic bone development adjacent to invasive synovial fibroblasts. Such ectopic endochondral ossification leads to the failure of bone repair and results in ankylosis. In this review, we describe the character of synovial fibroblasts toward the osteochondrogenic lineage and ectopic endochondral ossification in an inflammatory arthritis mouse model.

## Background

In rheumatoid arthritis (RA), a variety of histopathological features are observed, such as pannus formation due to hyperplasia of the synovial membrane with inflammation and bone erosion. In pannus, macrophage- and fibroblast-like synovial cells are classified as type A and type B synoviocytes, respectively. Macrophage-like synoviocytes originate from the bone marrow. On the other hand, fibroblast-like synoviocytes are derived from synovial membrane in the joints. In most patients with RA, the majority of synoviocytes in pannus are type B synovial cells (hereafter called synovial fibroblasts). Synovial fibroblasts express vimentin as a specific marker of mesenchymal cells and S100A4 (also known as fibroblast-specific protein 1, FSP1) as an epithelial-to-mesenchymal transition-related marker [1, 2]. However, S100A4 for example is expressed in immune cells including macrophages in degraded cartilage of patients with RA [3]. Podoplanin (Pdpn) was detected in the hyperplastic synovial lining layer and sub-lining layer in RA joints [2]. The expression of Pdpn was increased by stimulation with cytokines, including interleukin 1-β, IL-1β, tumor necrosis factor α, TNFα, and transforming growth factor β, TGFβ [4]. Synovial cells are primary fibroblast-like cells from amniotic membranes. Thus, these synovial fibroblasts are functionally equivalent to mesenchymal stem cells (MSCs) [5]. As MSC markers, Thy1 is expressed in a subpopulation of synovial cells [6]. Various in vitro studies have shown that synovial cells from the synovium are capable of differentiating into chondrocytes, adipocytes, and osteoblasts [7, 8]. Synovial fibroblasts also express osteochondrogenic markers such as type II collagen (ColII) [9]. There is no specific marker of synovial fibroblasts for use in histopathological analysis because of the variability of their features.

**Fig. 1:**
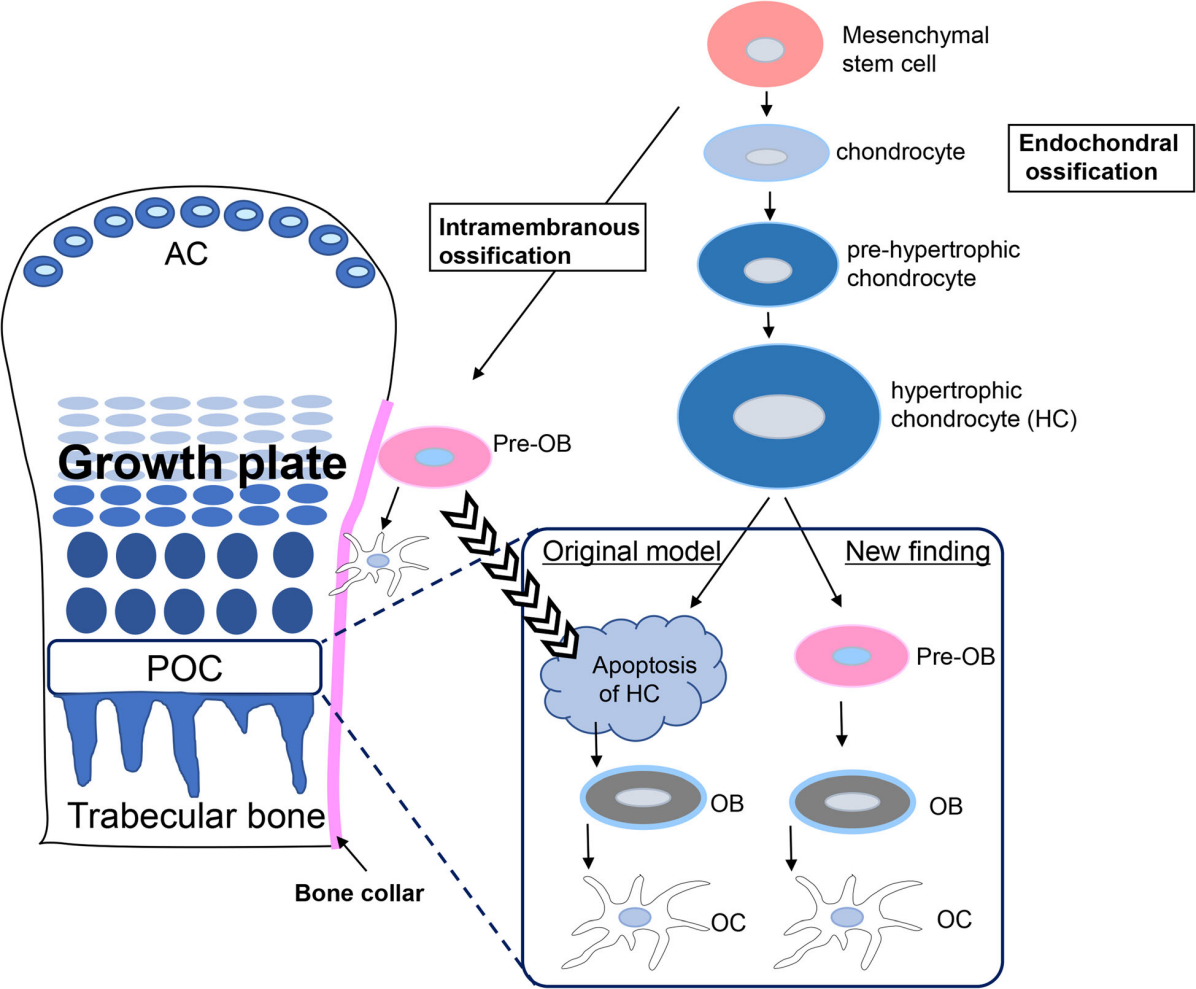
Fate of hypertrophic chondrocytes in the growth plate. In the primary ossification center, POC, hypertrophic chondrocytes enter apoptosis; it was the original theory for endochondral ossification in the growth plate. Recent data on the fate of hypertrophic chondrocytes in the growth plate are controversial because hypertrophic chondrocytes directly differentiate into osteoblasts in the POC. AC, articular chondrocyte: OB, osteoblast; OC, osteocyte

Osteoblasts are derived from MSCs from neural crest cells or mesodermal cells. MSCs directly differentiate into osteoblasts, which form a sheet-like structure bone and limb cortical bone, referred to as intramembranous ossification (Fig. 1) [10, 11]. On the other hand, the bone formation in the trunk and limb is called endochondral ossification, which involves the differentiation from MSCs into chondrocytes followed by hypertrophic chondrocytes. Hypertrophic chondrocytes mineralized matrix and enter apoptosis in the primary ossification center (POC). These apoptotic hypertrophic chondrocytes are replaced with pre-osteoblasts in the POC (Fig. 1) [1, 12]. Recent studies on endochondral ossification have revealed that hypertrophic chondrocytes differentiate directly into osteoblasts (Fig. 1). Because the regeneration of these normal bone formation is disordered in RA, bone erosion and osteoporosis are simultaneously observed as distinctive terminal symptoms of RA. Understanding the mechanisms of these disorders of bone regeneration, starting from synovial cell dysfunction, should lead to the discovery of therapeutic goal for synovial fibroblasts in RA.

## Pannus formation in arthritis model

Collagen-induced arthritis (CIA) has been investigated as an inflammatory joint-related form of arthritis by immunization using ColII with Freund’s complete and incomplete adjuvants [13]. However, most arthritis models, such as CIA, involve the induction of acute inflammatory arthritis and show bone destruction, but no ankylosis, and eventually weak osteoporosis. This is because osteophytes are often observed in the CIA mouse model, resulting in a slight decrease in the total bone mineral density in joints. Thus, in terms of bone formation, the CIA model resembles osteoarthritis rather than RA [14, 15].

**Fig. 2:**
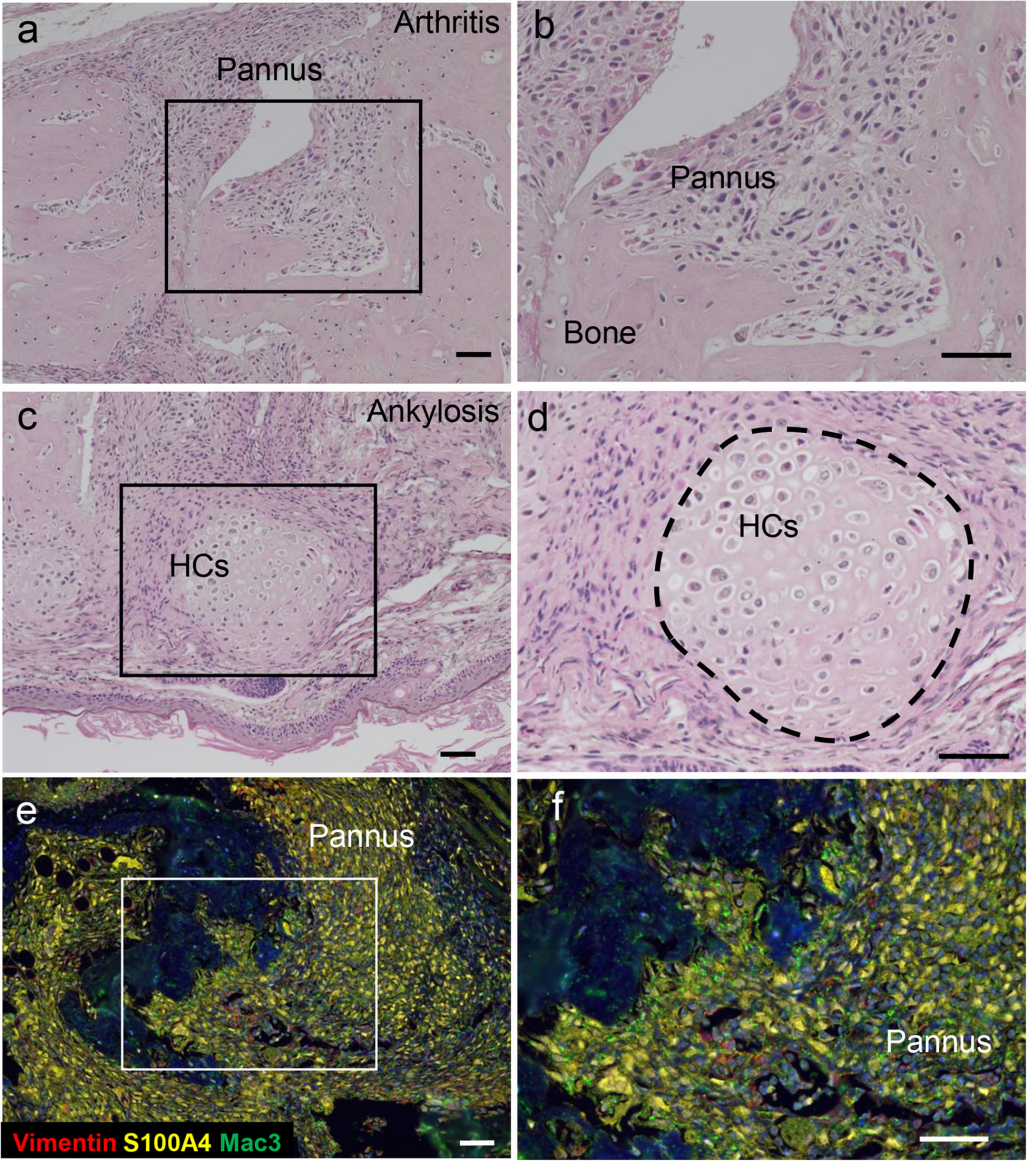
Pannus formation, bone destruction, and ankylosis in bColII-D1BC mouse [16]. **a–d** H&E staining for pannus formation and bone destruction (**a**, **b**) and ankylosis (**c**, **d**) in bColII-D1BC mouse joints. D1BC mice were injected with a low dose of bColII (0.02 mg/mouse). Following chondrogenesis, hypertrophic chondrocytes (HCs) were localized adjacent to invasive synovial fibroblasts in pannus in D1BC mouse joints. **e** and **f** Immunohistochemical staining of vimentin (red), S100A4 (yellow) ,and Mac3 (green) in bColII-D1BC mouse joints. Each box in a, c, and e represents a magnified area from each right panel. Scale bar = 50 μm

D1BC mouse (DBA/1J, B7.1 gene transcribed from the rat ColII promoter and enhancer) was established as a novel arthritis model [16]. In D1BC mouse, B7.1 is specifically expressed in chondrocytes because murine B7.1 is located between the ColII promoter and enhancer. D1BC mice do not show any arthritis spontaneously, but do develop severe and chronic inflammatory arthritis upon immunization with a low concentration of bovine ColII (bColII, 0.02 mg/mouse, less than 1/10 of the level in the conventional CIA method) [16]. Histopathological analysis of inflamed joints in bColII-induced D1BC (bColII-D1BC) mouse revealed severe pannus formation and bone destruction, followed by the emergence of chondrocytes, hypertrophic chondrocytes, and bone adjacent to hyperplastic synovial fibroblasts (Fig. 2a–d). Most synovial fibroblasts are S100A4-positive cells, and cells positive for the macrophage marker, Mac3 are in a minority in the pannus of bColII-D1BC mouse (Fig. 2e, f). Although infiltrated inflammatory lymphoid cells including macrophages and CD3-positive T cells are observed, these cells are also relatively minor populations compared with S100A4-positive synovial fibroblasts. These results suggest that most synovial cells are synovial fibroblasts in the pannus of bColII-D1BC mouse [16].

### *Runx2, Sox9,* and *Col10a1* triple positive synovial cells in inflamed joints

**Fig. 3:**
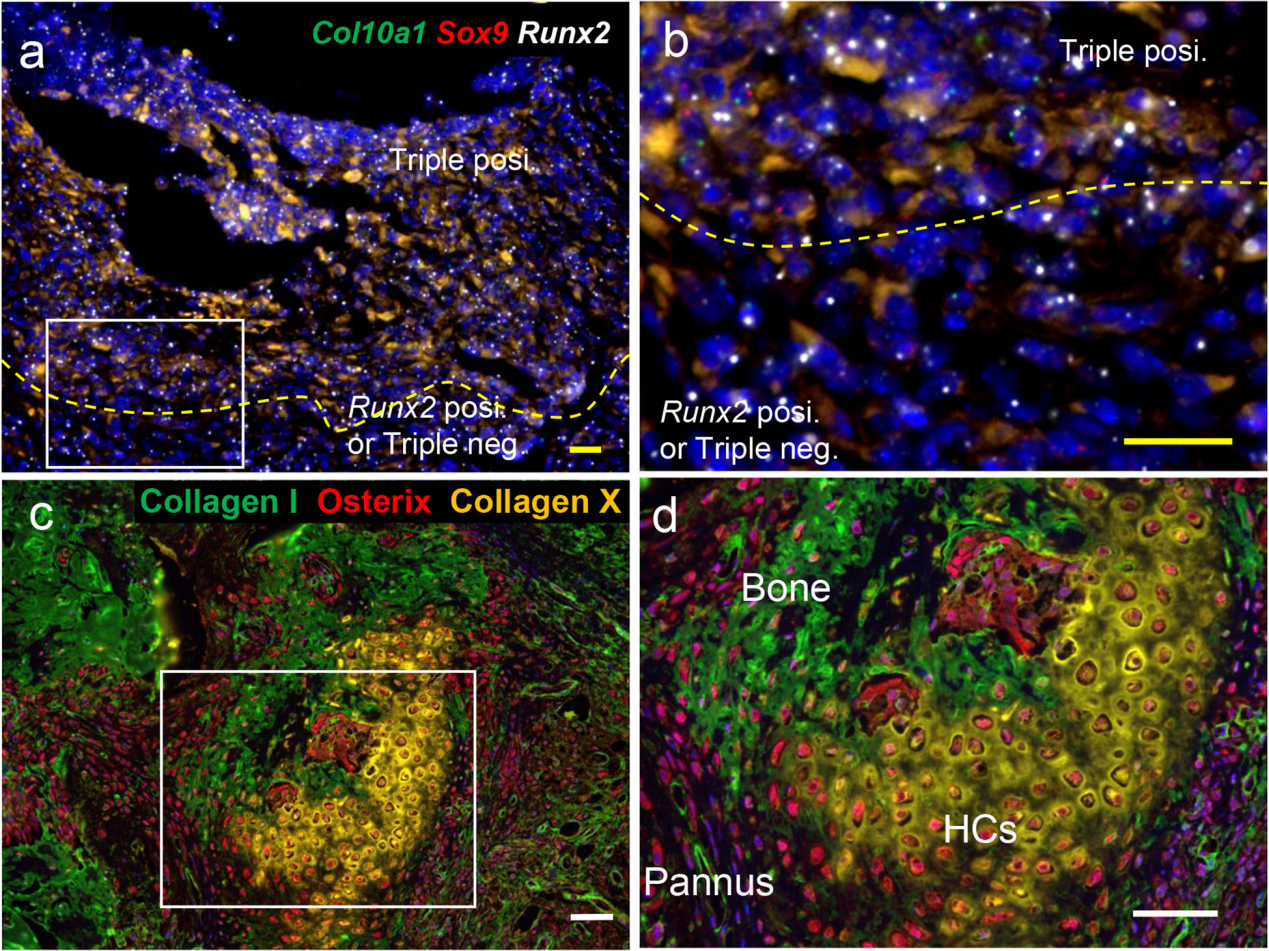
A subpopulation of SFs expressing *Runx2*, *Sox9*, and *Col10a1* in pannus retain competence as OCs via HCs [16]. **a, b** In situ hybridization was performed. *Sox9* (red), *Runx2* (white), and *Col10a1* (green) triple-positive synovial fibroblasts are present in pannus, along with *Runx2* single-positive and triple-negative ones. **c, d** Immunohistochemical staining was performed. Osteocytes express ColI (green), and hypertrophic chondrocytes (HCs) express ColX (yellow). Both proteins are detected as extracellular matrix. In addition to pannus, both bone (osteocytes) and HCs express osterix (red). Each box in a and c represents a magnified enlargement area in each right panel. Scale bar = 20 μm (yellow) and 50 μm (white)

Fibroblasts can generally differentiate into connective tissue cells including osteoblasts, chondrocytes, adipocytes, and smooth muscle cells. We studied whether synovial fibroblasts, which function as MSCs, are involved in subsequent osteochondrogenic differentiation in inflamed joints of bColII-D1BC mouse. To this end, many studies of bone remodeling during fracture repair and osteochondrogenic differentiation in growth plates have suggested various cell markers for immunostaining and in situ hybridization [17–19]. We performed in situ hybridization using the osteochondrogenic differentiation markers runt-related transcription factor 2 (*Runx2*), *Sox9*, a member of the SRY-related high-mobility group box transcription factor family and, *Col10a1* in synovial fibroblasts, hypertrophic chondrocytes and osteocytes of inflamed joints. Osteochondrogenic synovial fibroblasts in pannus were composed of at least three different subpopulations, which express (1) *Runx2*, *Sox9*, and *Col10a1*; (2) *Runx2* alone; and (3) none of these, referred to as a triple-negative state (Fig. 3a, b). Since these synovial fibroblasts express osterix (Osx, also known as SP7), which is one of the master regulators of osteoblast differentiation, they are in the osteochondrogenic lineage (Fig. 3c, d) [19]. On the other hand, type X collagen (ColX) as a component of the cartilage matrix is not detected in these synovial fibroblasts of pannus; therefore, they are at the stage of relatively immature hypertrophic chondrocytes such as pre-hypertrophic chondrocytes [16]. Because normal synovial membrane in the joint does not express these osteochondrogenic markers, synovial fibroblasts in synovium tend to be differentiated into the osteochondrogenic lineage. This may cause bone dysfunction such as ankylosis due to the failure of regeneration in the joint.

### The mechanisms of endochondral ossification

**Fig. 4:**
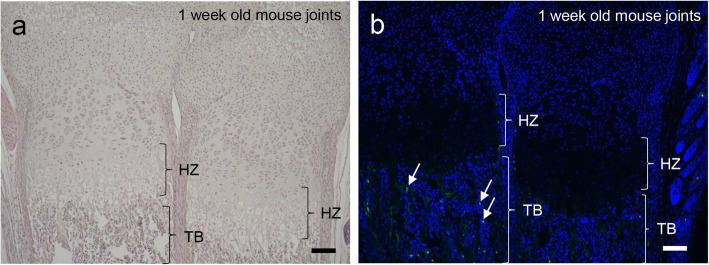
Minor population of apoptotic cells in hypertrophic chondrocytes near the growth plate in the joint. **a** H&E staining of 1-week-old mouse joints. **b** TUNEL assay in 1-week-old mouse joints. Apoptotic cells are indicated by arrows in trabecular bone (TB). There are no apoptotic cells in the hypertrophic zone (HZ). Scale bar = 100 μm

In endochondral ossification, MSCs differentiate into hypertrophic chondrocytes via chondrocytes in the growth plate at the joint. According to the original theory of endochondral ossification, osteoblast precursors from cortical bone in intramembranous ossification migrate into the primary ossification center (POC) where hypertrophic chondrocytes enter apoptosis (Fig. 1) [11]. Thus, hypertrophic chondrocytes were replaced by Osx-positive osteoblast precursor cells. However, recent studies have demonstrated that hypertrophic chondrocytes do not necessarily need to enter apoptosis. Because hypertrophic chondrocytes express anti-apoptotic protein B-cell lymphoma 2, Bcl2, they can differentiate into osteoblasts (Fig. 1) [20]. In fact, hypertrophic chondrocytes secrete bone matrix, including alkaline phosphatase, osteocalcin, osteopontin, and bone sialoprotein, during bone development [21, 22]. An in vitro study demonstrated that hypertrophic chondrocytes from chick embryo tibiae mineralize and differentiate into osteoblast-like cells [23]. Thus, using the cell lineage tracing technique, the fate of hypertrophic cells in developmental regulation of the growth plate was revealed. All these findings suggest that hypertrophic chondrocytes are involved in endochondral bone formation [12, 19, 22]. We also detected only a minor population of apoptotic hypertrophic chondrocytes in newborn mouse, suggesting that some of the hypertrophic chondrocytes differentiated directly into osteoblasts (Fig. 4a, b).

In endochondral ossification, Sox9 is one of the master transcription factors. Sox9 binds to the enhancer region of the Col2a1 gene, which regulates the development of chondrocytes [24]. Sox9 is a negative regulator of the expression of ColX during osteochondrogenic differentiation in the growth plate. When chondrocytes mature and differentiate into pre-hypertrophic chondrocytes, the expression of *Col10a1* is upregulated by the downregulation of *Sox9* [25]. Chondrocytes differentiate into hypertrophic chondrocytes that produce ColX during their maturation. The role of ColX is still unknown, but might be correlated with calcification [26]. Runx2 is an essential transcription factor for regulation of the promoter regions of osteocalcin, type I collagen (ColI), and osteopontin in osteoblasts. Osx is also a transcription factor for bone differentiation and exerts regulatory function downstream of Runx2 for cartilage matrix ossification and degradation. The mature osteoblasts are embedded into bone matrix and differentiate into osteocytes.

### Bone erosion by MMPs and cathepsins

Bone erosion is one of the major symptoms of RA. A larger volume of the synovial membrane increases the risk of erosive joint destruction [27]. Severe joint inflammation also causes bone destruction and reduces bone mineral density in a D1BC-related RA model, D1CC mouse [14]. Similar bone disruption was also seen in D1BC mouse [16]. One of the major reasons for the bone destruction in RA is that matrix metalloproteinases (MMPs) and cathepsins from osteoclasts and/ or synovial fibroblasts cleave bone matrix including ColI and proteoglycans [28]. A large number of osteoclasts are observed at the synovium-bone interface in RA joints by TRAP staining [29]. Rheumatoid synovial fibroblasts, which express receptor activator of nuclear factor kB ligand (RANKL), induce osteoclastogenesis for peripheral blood mononuclear cells, PBMCs in the presence of 1,25(OH)_2_D_3_ [30]. In addition, bone destruction is triggered not only by osteoclasts but also by synovial cells in pannus [31]. This is because synovial fibroblasts secrete MMPs such as MMP1, 3, 9, 10, and 13, and accelerate bone erosions in the joints [28]. Cathepsins K and L are also expressed in RA synovial fibroblasts and contribute to the degradation of bone matrix, including ColI, II IX, and XI and proteoglycans [28, 32, 33]. We detected increased mRNA expression of MMPs such as *Mmp2*, *Mmp3*, *Mmp10*, and *Mmp13*, and cathepsins such as *Ctsa*, *Ctsk*, and *Ctsl* in joint lavage fluid from bColII-D1CC × D1BC double transgenic mice by RNA array analysis (data not shown). These results suggest that RANKL and osteolytic proteinases such as MMPs and cathepsins from RA synovial cells are major causes of bone destruction and excellent therapeutic targets.

### Differentiation from hypertrophic chondrocytes to osteocytes in pannus

**Fig. 5:**
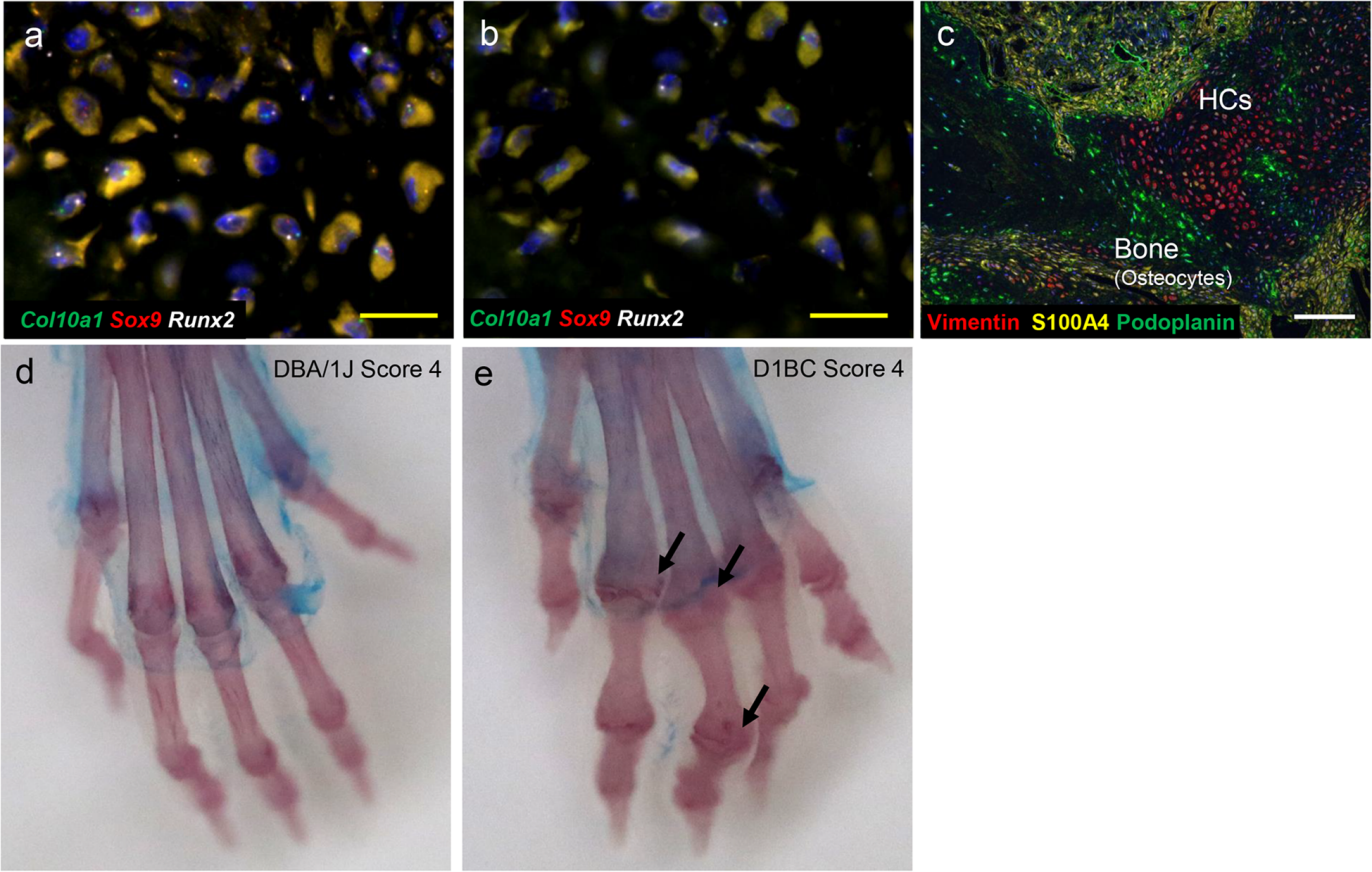
Hypertrophic chondrocytes express *Runx2*, *Sox9*, *and Col10a1.* In situ hybridization was performed [16]. **a, b***Sox9* (red), *Runx2* (white), and *Col10a1* (green) mRNA signals in hypertrophic chondrocytes (**a**) and osteocytes (**b**) in bColII-D1BC inflamed joints. **c** In pannus, OCs in bone, HCs, and synovial fibroblasts express podoplanin (green), vimentin (red), and S100A4 (yellow), respectively. Scale bar = 20 μm (yellow) and 50 μm (white). **d, e** Joint space narrowing, erosion, and osteoporosis in bColII-D1BC mice. D1BC and DBA/1J mice were injected with a low-dose of bColII to induce joint inflammation. Whole-mount bone and cartilage staining with alizarin red S and alcian blue, for bone (red) and cartilage (blue), respectively. All mouse limbs at 1 year after the first immunization. No ankylosis in CIA-induced DBA/1J mouse (**d**). Note, the complete destruction of all joints (ankylosis, allow) and shortening of the phalanges in bColII-D1BC mouse joints (**e**)

Bony ankylosis was seen in bColII-D1BC mice; this aberrant ossification might reflect a failure of hypertrophic cartilage remodeling. *Sox9* expression is suppressed in normal hypertrophic chondrocyte differentiation [25], but hypertrophic chondrocytes in inflamed joints express *Runx2*, *Sox9*, *Col10a1*, ColX, and Osx (Figs. 3c and 5a). Osteocytes adjacent to hypertrophic chondrocytes still express *Runx2*, but not *Sox9*, *Col10a1*, Osx, and ColI (Figs. 3c and 5b). Pdpn is also expressed in osteocytes to enhance mineralization of the extracellular matrix (Fig. 5c) [34]. Thus, although the synovial fibroblasts might be associated with osteochondrogenesis, these de novo chondrogenic and osteogenic cells could come from proliferative synovial fibroblasts in pannus and not from articular cartilage or other chondrogenic cells in the inflamed joints.

### Bony ankylosis in bColII-D1BC mouse joints similar to RA

As a result of severe polyarthritis, failure of joint regeneration leads to bony ankylosis in bColII-D1BC mouse. Such stiff ankylosis-affected joints do not bend and stretch due to the bone-to-bone connections, but no ankylosis in CIA-induced DBA/1J mouse (Fig. 5d, e). Interestingly, despite the observation of aberrant bone formation in bColII-D1BC mouse, bone mineral density was reduced in the joints, similar to the findings in patients with RA [16]. Therefore, aberrant bone formation results in dysfunction of the joints and D1BC mouse are useful to study osteochondrogenic differentiation and aberrant bone formation.

## Conclusion

**Fig. 6:**
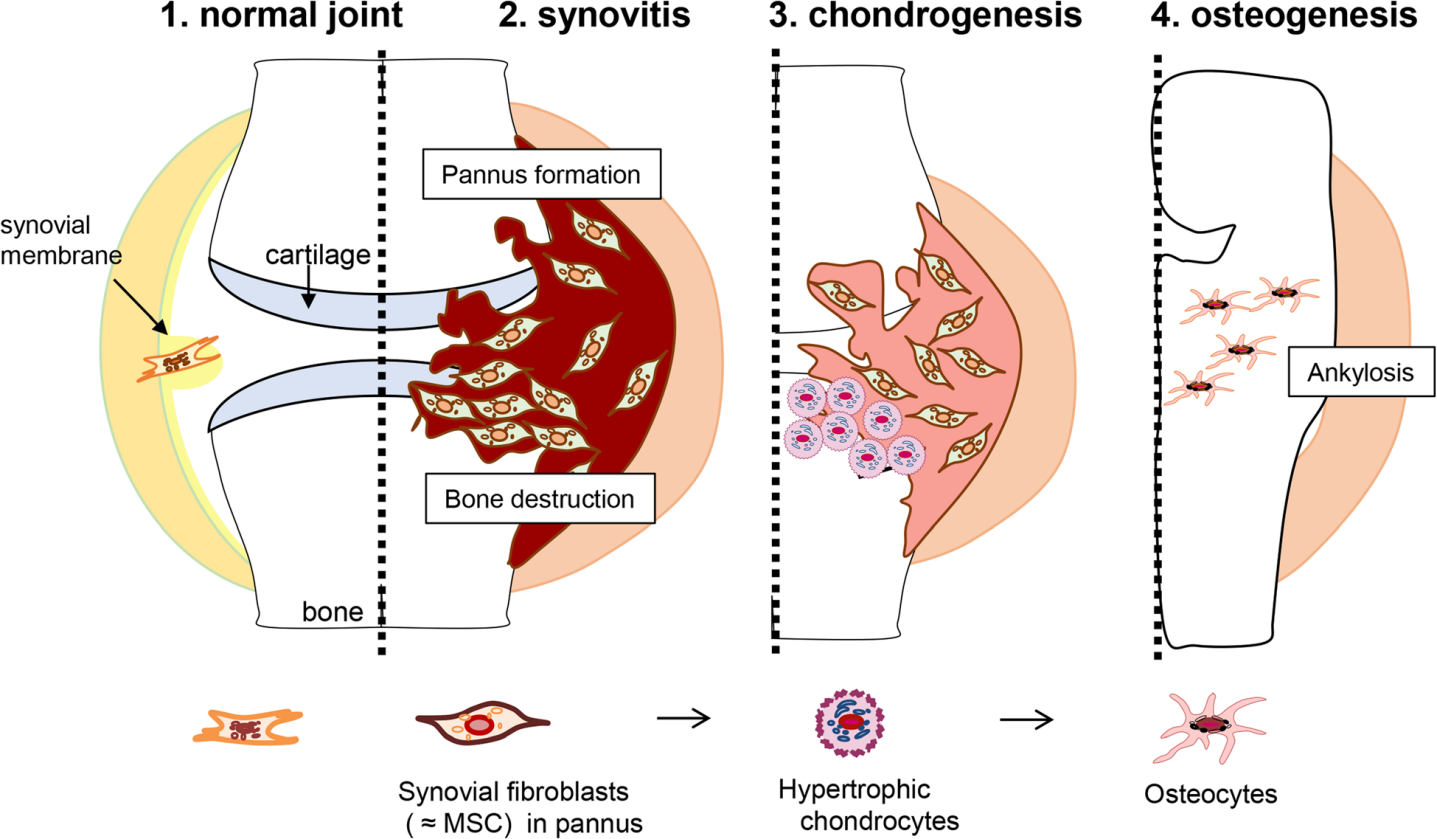
Schematic representation of the stages of RA in D1BC mouse model [16]. 1: Synovial membranes, which consist of one to three cell layers, are the origin of synovial fibroblasts. 2: Joint inflammation induces aggressive proliferation of synovial fibroblasts, followed by bone erosion and destruction in the inflamed joint. Synovial fibroblasts share cell lineage markers of MSCs with pre-hypertrophic chondrocytes in bColII-D1BC mice. Because the most definitive feature of synovial fibroblasts in pannus is the expression of *Col10a1* mRNA, but not its transcribed protein, ColX, these are defined as pre-hypertrophic chondrocytes. Thus, a subpopulation of synovial fibroblasts (*B7.1*, *Col2a1*, *Runx2*, *Sox9*, *Col10a1*, and Osx) differentiates into ColX-negative hypertrophic chondrocytes. 3: Synovial fibroblasts differentiate into hypertrophic chondrocytes by hypertrophic cartilage remodeling in the process of endochondral bone formation. 4: When hypertrophic chondrocytes terminally differentiate into osteochondrocytes, the expression of ColI is observed instead of ColX. A failure of cartilage remodeling leads to aberrant bone formation by ossified chondrocytes, resulting in bony ankylosis

Note that bColII-D1BC mice showed ankylosis at the end of disease progression with extra mineralization in parallel with osteoporosis; this de novo osteochondrogenesis might reflect a failure of remodeling of the bone development in inflamed joint [16]. We identified osteocytes adjacent to hypertrophic chondrocytes (*Col2a1*, *B7.1* as a transgene in D1BC mouse, *Runx2*, *Sox9*, and *Col10a1*), although these cells still expressed *B7.1*^low^, Osx, podoplanin, and ColI, but no detectable levels of *Col10a1* or ColX. Figure 6 shows a schematic model depicting the histopathological features in inflammatory arthritis-induced D1BC mouse. D1BC mouse exhibits chronic, slow disease progression, which facilitates study such as time-lapse analysis of pannus formation and osteochondrogenic differentiation. Synovial fibroblasts, but not synovial macrophages, function as major effector cells, which produce chemokines and cytokines along with matrix metalloproteinases [35, 36]. In inflammatory arthritis-induced D1BC mouse, a subpopulation of these aggressive synovial fibroblasts in synovitis is in the osteochondrogenic lineage. These synovial fibroblasts might differentiate into osteocytes via hypertrophic chondrocytes, resulting in the development of ankylosis by endochondral ossification. Understanding this inappropriate osteochondrogenic differentiation in RA can lead to the development of therapeutic approaches for joint remodeling and the development of ankylosis.

## Data Availability

The datasets used and/or analyzed during the current study are available from the corresponding author on reasonable request.

## Abbreviations

CIA: Collagen-induced arthritis
ColII: Type II collagen
D1BC: DBA/1J, B7.1 gene transcribed from the rat type II collagen promoter and enhancer
MMPs: Matrix metalloproteinases
MSCs: Mesenchymal stem cells
Pdpn: Podoplanin
POC: Primary ossification center
RANKL: Receptor activator of nuclear factor kB ligand
RA: Rheumatoid arthritis
Runx2: Runt-related transcription factor 2
